# Effect of Different Polishing Systems on the Surface Roughness of 3D-Printed Resins

**DOI:** 10.3390/ma19183831

**Published:** 2026-09-09

**Authors:** Nilge Sarimehmetoglu, Huseyin Simsek

**Affiliations:** 1 Department of Pediatric Dentistry, Faculty of Dentistry, Giresun University, 28200 Giresun, Turkey; 2 Department of Pediatric Dentistry, Faculty of Dentistry, Ordu University, 52200 Ordu, Turkey; dr.huseyinsimsek@gmail.com

**Keywords:** 3D-printed resin, dental materials, finishing, polishing, surface roughness

## Abstract

**Highlights:**

**What are the main findings?**

- Finishing and polishing systems significantly affected Ra values.
- All systems reduced surface roughness in 3D-printed crown resins.
- The resin type did not significantly influence roughness outcomes.

**What are the implications of the main findings?**

- The choice of polishing system affects the final surface quality.
- Finishing and polishing are essential after 3D crown resin printing.
- Proper polishing may reduce biofilm retention on printed restorations.
- Clinicians should consider polishing protocol selection after printing.

**Abstract:**

Background: This in vitro study evaluated the effect of different finishing and polishing systems on the surface roughness of two 3D-printed crown resins. Methods: Eighty disc-shaped specimens were produced from two 3D-printed crown resins, Saremco Print Crowntec and VarseoSmile Crown Plus. Specimens were divided into four groups according to the finishing and polishing protocol: Lucida, TwistDia, OptiShine, and OptiGlaze. Surface roughness (Ra) was measured before and after finishing and polishing using a non-contact optical profilometer. Data were analyzed using ART ANOVA (*p* < 0.05). Results: All polishing protocols significantly reduced surface roughness, with the overall median Ra decreasing from 0.198 µm before polishing to 0.101 µm after polishing (*p* < 0.001). Among the tested protocols, OptiGlaze showed the lowest post-polishing median Ra values for both resin materials, whereas Lucida showed the lowest overall median Ra value across measurement times. Resin type had no significant effect on Ra, whereas the polishing system significantly influenced surface roughness. Pairwise comparisons revealed significant differences between OptiGlaze and OptiShine, OptiGlaze and TwistDia, and Lucida and TwistDia (*p* < 0.05). All post-polishing Ra values remained below the clinically relevant threshold of 0.2 µm. Conclusions: The surface quality of the tested 3D-printed crown resins was determined primarily by the polishing protocol rather than the resin type. Although all protocols provided clinically acceptable surfaces, the optimal polishing approach may depend on the resin–polishing system combination.

## 1. Introduction

Aesthetic expectations are important in dentistry not only for adults but also for pediatric patients. While there is a considerable amount of literature available on aesthetic dentistry for adult patients, information on this subject is limited for pediatric patients [1]. In the treatment of dental caries in pediatric patients, it is important to choose the most appropriate restorative method. For primary teeth, pediatric crowns are durable restorations with long-term clinical success, particularly following endodontic treatment. Prefabricated pediatric crowns are available in a variety of sizes and forms. The oldest and most commonly used type is the stainless steel crown (SSC). However, SSCs do not meet aesthetic expectations because of their gray color. As an alternative to SSCs, pediatric zirconia crowns (PZCs), which mimic the natural tooth color, have been developed [2]. PZCs are also prefabricated crowns and are restorative materials that satisfy aesthetic demands while offering durability and high wear resistance. Nevertheless, their potential to cause wear on the opposing tooth and the difficulties encountered in adapting them to the tooth because of their rigid structure have been reported as clinical disadvantages [3].

Advances in digital technologies have significantly impacted clinical and laboratory practices in dentistry [4]. Especially with three-dimensional (3D) printing technology, it has become possible to produce dental restorations in a short time and at a lower cost [4,5]. Resin-based hybrid ceramics used in 3D printing are materials with elastic moduli close to dentin and are preferred for primary tooth restorations. Their high fracture resistance and superior performance in withstanding occlusal forces also make 3D-printed hybrid resins a suitable option for pediatric restorations [3].

3D-printed resins have been reported to exhibit similar physical properties to composite resins. As with composite resins, finishing and polishing procedures are essential steps for achieving optimal surface properties in 3D-printed resins after fabrication. Although no standardized protocol specifically for the finishing and polishing of 3D-printed resins has yet been established, manufacturers recommend procedures similar to those used for composite resins [6].

The surface properties of restorative materials are closely related to clinical conditions such as discoloration, plaque retention, recurrent caries, and periodontal health [7,8,9]. Achieving a smooth restoration surface increases the long-term success of restorations. Surface roughness of dental materials below 0.2 µm has been widely suggested as a clinically significant threshold for bacterial adhesion [10]. A smooth surface can be obtained through appropriate finishing and polishing (F&P) procedures. For this purpose, a variety of finishing and polishing systems with different characteristics have been developed [11]. A wide range of single-step and multi-step polishing systems has been introduced to the market, including aluminum oxide-coated abrasive discs, diamond-impregnated silicone or rubber points and discs, and diamond-containing polishing pastes [7,11,12].

Although additive manufacturing enables the efficient fabrication of definitive restorations with complex geometries, the layer-by-layer production process and subsequent post-processing procedures may result in surface irregularities. These irregularities may require additional finishing, polishing, or surface-coating procedures before clinical use. Moreover, differences in resin matrix composition, filler characteristics, printing technology, and post-curing conditions may influence the response of 3D-printed materials to surface treatment. Previous studies have reported inconsistent findings regarding the effects of surface treatment procedures on the surface roughness of permanent 3D-printed crown resins. While some studies have reported lower surface roughness following mechanical polishing, others have demonstrated comparable or lower Ra values after coating based surface treatments [13,14,15]. Moreover, the effectiveness of individual polishing systems appears to vary according to the resin material evaluated. These differing results suggest that surface treatment effectiveness may be dependent on both the material and the protocol, and that no single approach can yet be considered universally optimal. Consequently, a surface treatment that is effective for one 3D-printed resin may not produce an equivalent surface quality in another material [15,16]. A comparative evaluation of different finishing and polishing protocols, which have previously been examined separately but not within the same experimental framework, may provide a valuable contribution to the literature. Therefore, this study aims to evaluate the effect of systems with varying numbers of application steps, abrasive configurations, and surface treatment mechanisms on the surface roughness (Ra) of 3D resins. Accordingly, the null hypothesis (H_0_) of the study was that: (1) the type of 3D-printed crown resin would not affect surface roughness values; (2) there would be no significant difference in surface roughness values among the different F&P systems.

## 2. Materials and Methods

**Figure 1:**
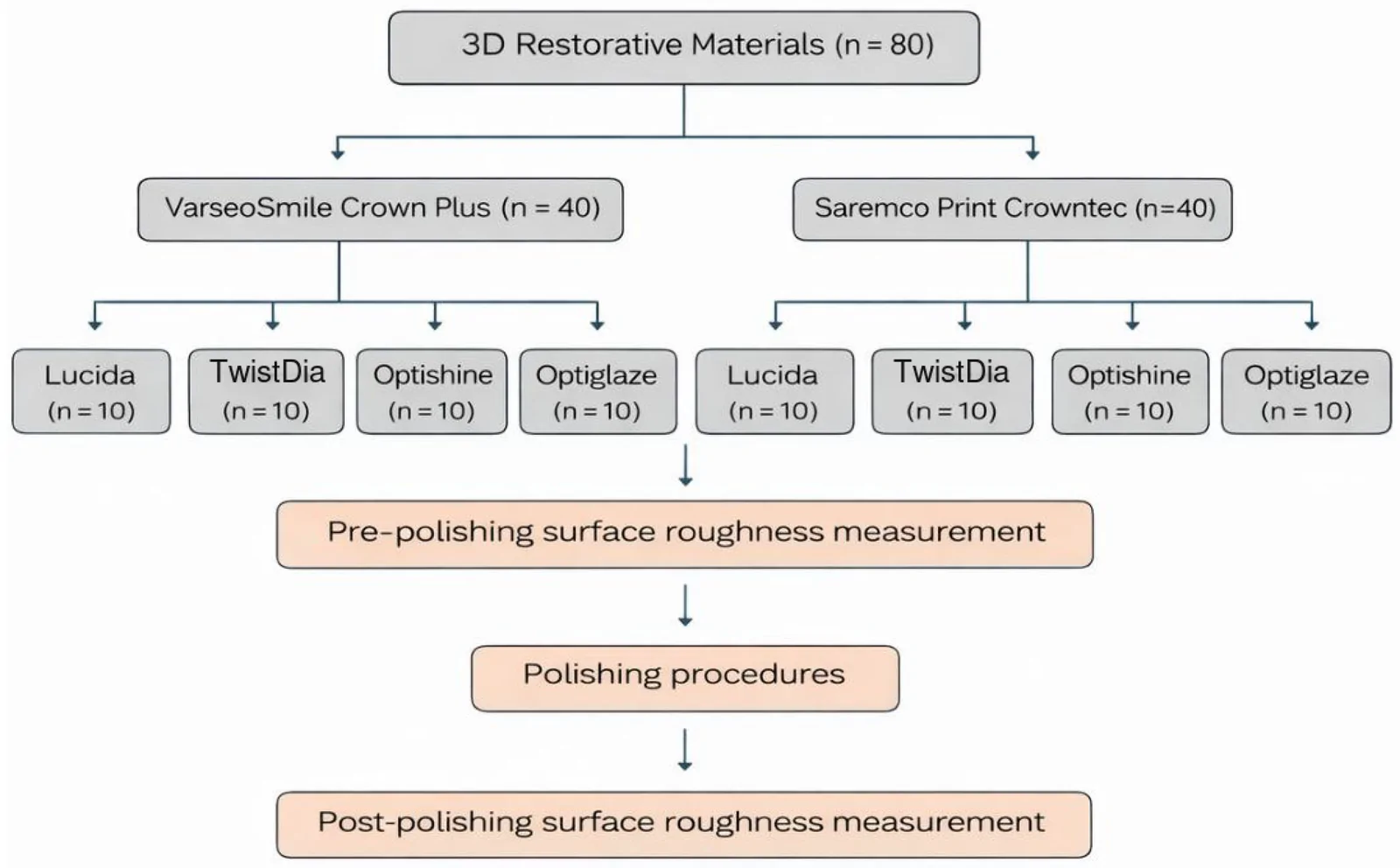
Flow chart of the study.

In this study, two different 3D printing resins (VarseoSmile Crown Plus, BEGO, Bremen, Germany and Saremco Print Crowntec, Rebstein, Switzerland) were used. The flow chart of the study is shown in Figure 1. Sample size was calculated using G*Power software (version 3.1.9.6, Düsseldorf, Germany) with 95% power and an error rate of 0.05 and effect size of f = 0.40 [17]. Accordingly, to ensure the reliability of the study results, the study was conducted with a total of 80 samples, with n = 10 samples per group. Information regarding the 3D printing resins used in the study is shown in Table 1.

The specimens were digitally designed in disc form (10 mm diameter × 3 mm thickness) using Exocad 3.1 software (Exocad, Darmstadt, Germany). The design data were converted into Standard Tessellation Language (STL) file format. The STL files were then transferred to a 3D printer (SprintRay Pro S, Los Angeles, CA, USA). A layer thickness of 50 μm was selected in accordance with the manufacturers’ recommended printing settings. The production angle was set to 0°. First, 40 disc-shaped specimens were fabricated using VarseoSmile Crown Plus resin (VSC); subsequently, 40 discs were produced on the same device using Saremco Print Crowntec resin (SPC).

Post-curing was performed using a curing device (SprintRay NanoCure, Los Angeles, CA, USA) at 385 nm for 3 min. Washing procedures were performed using an ultrasonic bath device (Phrozen Ultrasonic Cleaner; Phrozen Inc., Hsinchu, Taiwan) at 3 min for each group, with a 96% ethanol solution. Each resin group (VSC and SPC) was then divided into four subgroups for F&P procedures (n = 10). The finishing and polishing systems evaluated in the study were Lucida (DiaShine), Twist Dia (Kuraray), Optishine (Kerr), and Optiglaze (GC) (Table 2).

The initial surface roughness (Ra) values of all specimens were measured and recorded using a 3D non-contact optical profilometer (Profilm3D^®^, Filmetrics/KLA Instruments, San Diego, CA, USA). Surface roughness was evaluated using the line roughness mode of the optical profilometer. The arithmetic mean roughness (Ra) was calculated according to ISO 4287 [18] over an evaluation length of 839.2 µm. The cutoff wavelength was set as λc = 80 µm. Measurements were taken at three different points on each sample, and the average of these measurements was recorded as the initial value in micrometers (μm) for that sample.

All surface treatment procedures were performed by a single operator. The surface treatment protocols applied to the groups were as follows:

Group 1: Optiglaze (GC, Tokyo, Japan), a nano-filled light-cured surface coating material, was applied to the specimens in a single layer using a microbrush and polymerized for 3 min in a laboratory curing unit.

Group 2: Lucida (Diashine, Italy), a diamond-containing polishing paste system, was applied to the samples with a star-shaped felt brush at 5000 rpm for 1 min.

Group 3: Polishing was performed with a silicon carbide-based brush (Optishine, Kerr, Brea, CA, USA) at 5000 rpm for 1 min.

Group 4: TwistDia (Kuraray, Hattersheim am Main, Germany), a two-stage diamond-impregnated spiral system, was applied to the samples at 5000 rpm for 30 s per disc. First, a dark blue pre-polishing wheel with 14 µm grit was used, followed by a light blue high-gloss polishing wheel with 10 µm grit.

After the finishing and polishing processes were completed, all samples were stored at 37 °C for 24 h. Then, surface roughness values (Ra) were measured again using the same measurement protocol.

The data were analyzed using R software version 4.4.1. The normality of the data distribution was assessed using the Shapiro–Wilk test. Variables that did not show a normal distribution were compared according to the factors of 3D resin, polishing system, and time (before/after polishing) using aligned rank transform (ART) ANOVA with the ARTool package (R version 4.4.1, Vienna, Austria). This approach enables the nonparametric evaluation of both main and interaction effects in factorial designs. Multiple comparisons were performed with Bonferroni correction.

## 3. Results

In this study, surface roughness values were compared considering two different 3D resin materials (VSC and SPC), four different finishing and polishing systems (Lucida, TwistDia, Optishine and Optiglaze), and two time points (before polishing/after polishing) (Table 3).

Surface roughness values did not show significant differences according to 3D resin material, regardless of polishing system and time factors. The median value of SPC was found to be 0.148 µm, while the median value of VSC was 0.156 µm (Table 4).

**Figure 2:**
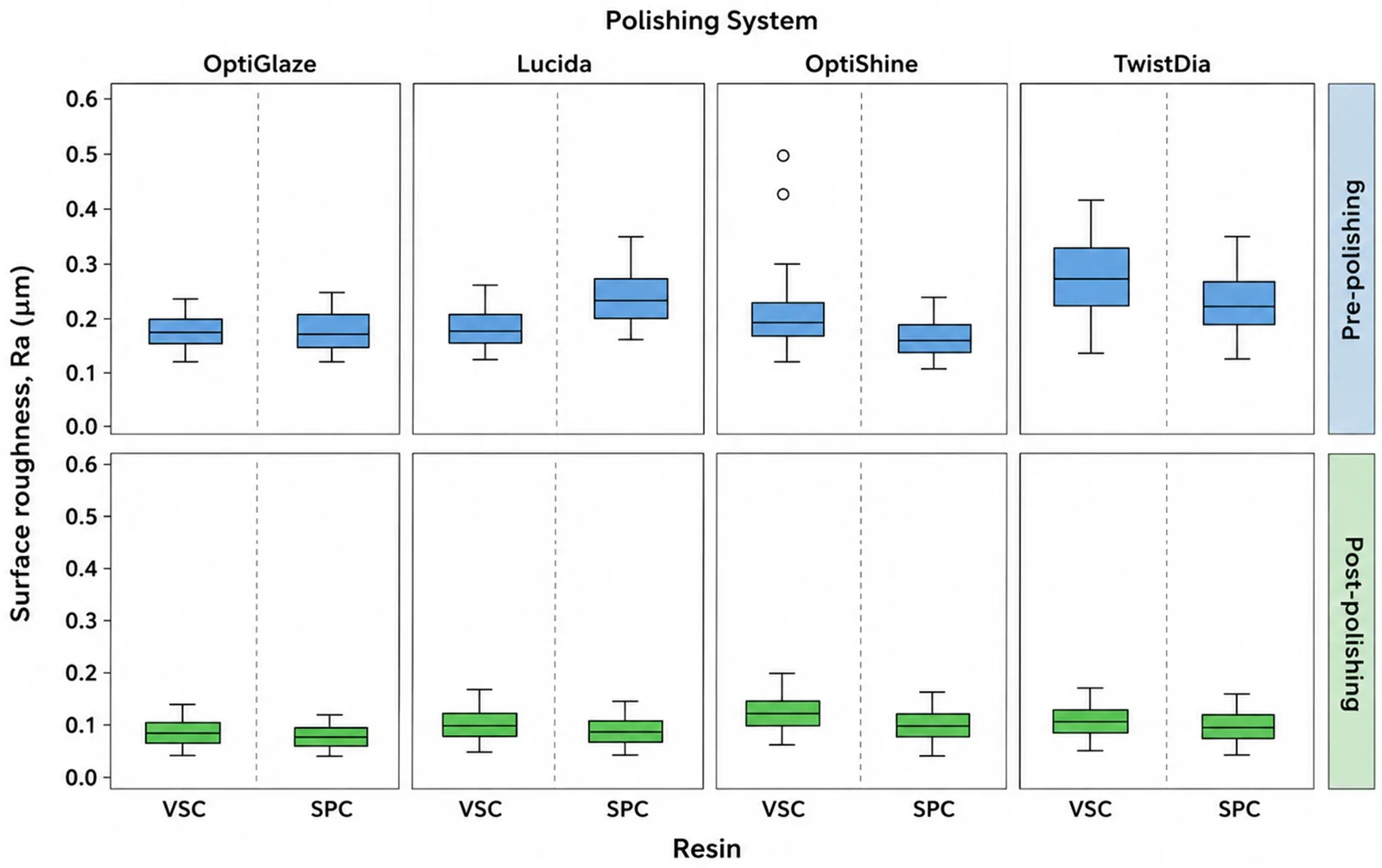
Surface roughness values of the resins according to polishing system at pre-polishing and post-polishing. Vertical dashed lines separate the resin groups (VSC and SPC), while open circles indicate outliers.

Independent of the resin material and time factors, the polishing systems showed a significant effect on surface roughness (*p* < 0.001). The mean surface roughness values according to polishing system were 0.153 µm for Optiglaze, 0.142 µm for Lucida, 0.150 µm for Optishine, and 0.159 µm for Twist Dia. In the multiple comparisons, significant differences were found between Optiglaze and Optishine, Optiglaze and Twist Dia, and Lucida and Twist Dia (Figure 2).

When the effect of the time factor on surface roughness was evaluated, surface roughness values were found to decrease significantly after the polishing procedures (*p* < 0.001). The mean surface roughness value was 0.198 µm before polishing and 0.101 µm after polishing.

A significant resin type × polishing system × treatment time interaction was detected (*p* = 0.002, partial η*p*^2^ = 0.179), indicating that the pattern of change in Ra from pre- to post-polishing differed according to the resin and polishing system combination. However, no specific pairwise comparison remained significant after Bonferroni adjustment. Surface roughness decreased numerically in all combinations. In SPC, the pre- and post-polishing median Ra values were 0.184 and 0.065 µm for OptiGlaze, 0.236 and 0.088 µm for Lucida, 0.172 and 0.116 µm for OptiShine, and 0.232 and 0.126 µm for TwistDia, respectively. In VSC, the corresponding values were 0.183 and 0.077 µm, 0.179 and 0.101 µm, 0.215 and 0.133 µm, and 0.283 and 0.113 µm.

When ΔRa values were analyzed separately within each resin, no significant difference was observed among the surface treatment systems for SPC (*p* = 0.067). For VSC, an overall significant difference was detected among the systems (*p* = 0.048); however, none of the pairwise comparisons remained significant after Holm adjustment (all adjusted *p* > 0.05). Numerically, the greatest median ΔRa was observed with Lucida for SPC (0.139) and TwistDia for VSC (0.142).

**Figure 3:**
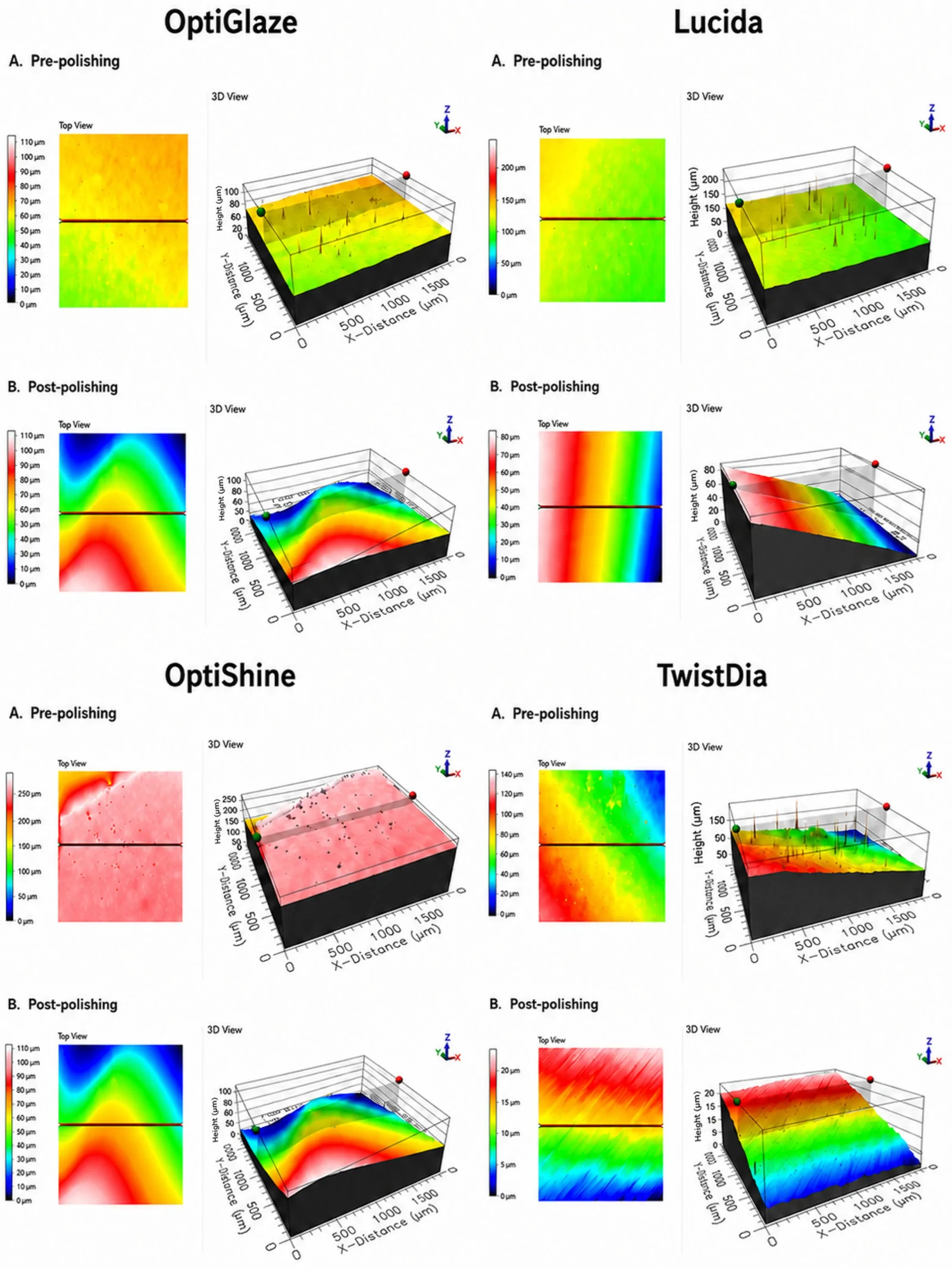
Representative three-dimensional optical profilometry images of VSC specimens before and after finishing and polishing. (The exact Ra values corresponding to the representative profilometry images were as follows: OptiGlaze Pre-polishing: 0.1818 µm, Post-polishing: 0.0781 µm. Lucida Pre-polishing: 0.1639 µm, Post-polishing: 0.1049 µm. OptiShine Pre-polishing: 0.2201 µm Post-polishing: 0.164 µm. TwistDia Pre-polishing: 0.2851 µm, Post-polishing: 0.1013 µm).

**Figure 4:**
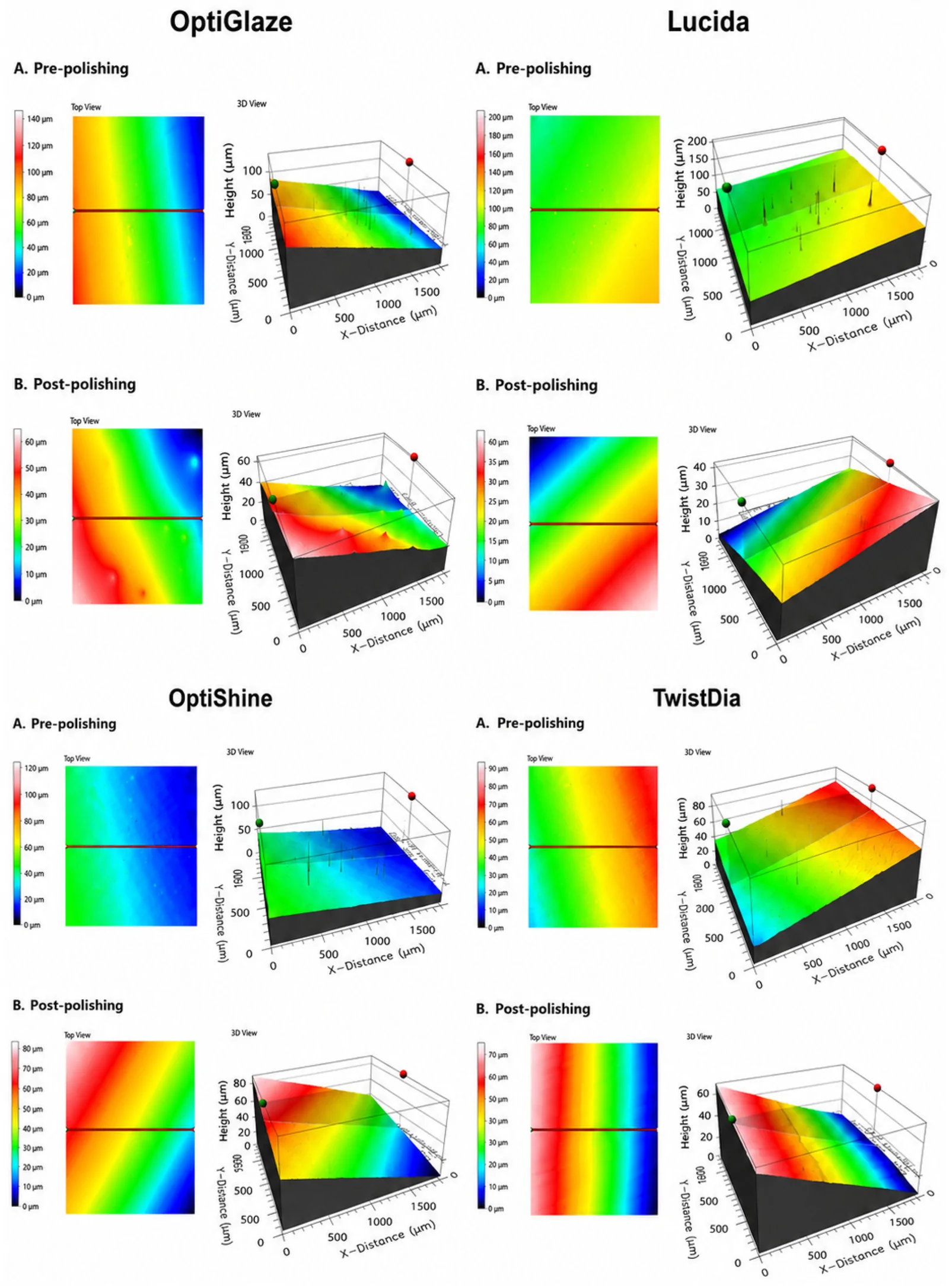
Representative three-dimensional optical profilometry images of SPC specimens before and after finishing and polishing. (The exact Ra values corresponding to the representative profilometry images were as follows: OptiGlaze Pre-polishing: 0.1881 µm, Post-polishing: 0.0638 µm. Lucida Pre-polishing: 0.2755 µm, Post-polishing: 0.0725 µm. OptiShine Pre-polishing: 0.1763 µm Post-polishing: 0.1227 µm. TwistDia Pre-polishing: 0.2271 µm, Post-polishing: 0.114 µm).

Representative 3D optical profilometry images were consistent with the quantitative findings. Representative samples from three-dimensional optical profilometry images were selected from those samples with Ra values closest to the median value of the respective experimental group. The images are presented for qualitative representation of surface morphology (Figure 3 and Figure 4).

## 4. Discussion

Due to the nature of the 3D-printing process, layer-by-layer fabrication may result in stair-step defects on the final restoration surface, which can increase the initial surface roughness [19,20]. Therefore, finishing and polishing procedures become even more clinically relevant for restorations fabricated by 3D printing. In the present study, the effects of different polishing systems on surface roughness of specimens fabricated from two different 3D-printing resins used for permanent restorations were investigated. The findings demonstrated that the Ra values of the 3D-printed resins varied significantly depending on the finishing and polishing system used. Therefore, the first null hypothesis could not be rejected because no significant main effect of resin type was identified. The second null hypothesis was rejected because surface roughness differed significantly among surface treatment systems.

Surface roughness of restorative materials is clinically important in terms of plaque retention, discoloration, preservation of surface gloss, and long-term aesthetic success of the restoration. In the literature, an Ra value of 0.2 µm is widely accepted as a clinically significant threshold for bacterial accumulation; it is reported that falling below this value may not provide an additional reduction in bacterial retention [21]. Therefore, the fact that the median Ra values after all polishings in our study were in the range of 0.065–0.133 µm and below the 0.2 µm threshold suggests that all the protocols examined were able to create clinically acceptable smooth surfaces. However, the 0.2 µm value should not be considered an absolute biological safety limit, but rather a commonly used reference value that facilitates the clinical interpretation of surface roughness.

The significant reduction in surface roughness observed after polishing is consistent with the results of recent studies on permanent 3D-printed resins. Bayraktar et al. reported that different polishing systems reduced the initial surface roughness in VarseoSmile Crown Plus and Saremco Print Crowntec materials; however, the resulting surface quality varied depending on the material and polishing protocol [15]. Similarly, in another study comparing single, two, and multi-stage polishing systems, it was shown that two and multi-stage systems, especially those complemented with paste, reduced the Ra values of permanent 3D-printed resins to below 0.2 µm [16]. These findings support the idea that post-3D printing surface treatments are not merely an aesthetic step, but are necessary for eliminating surface irregularities caused by printing and post-processing.

In our study, the effect of resin type on surface roughness was not found to be significant. However, this result should not be interpreted as meaning that the surface behavior of the two materials is the same under all conditions. It is reported that the polishing response can be affected by variables such as material composition, filler content and distribution, polymer matrix, printing technique, and post-curing conditions [22]. Microstructural examinations performed on VarseoSmile Crown Plus revealed irregular filler distribution, layered macrostructure, and pores due to the printing process, which are among the factors that can explain why products in the same material class may respond differently to mechanical surface treatments [23].

One possible explanation for the observed low Ra values is that OptiGlaze is applied as a surface coating, unlike mechanical polishing systems. While mechanical polishing systems remove surface irregularities by abrasion, low-viscosity surface coatings can create a more homogeneous surface by covering or filling micro-pits and irregularities [24,25,26]. In a recent study comparing varnish, diamond-impregnated rubber polishing system, and brush-paste protocol on three 3D-printed crown materials, it was reported that varnish application resulted in the lowest initial roughness in all materials and that all processes kept Ra values below 0.2 µm [14]. This result supports the low post-polishing values obtained in the OptiGlaze groups in our study. However, surface coatings do not have the same mechanism as mechanical polishing, and the integrity of the coating layer may be compromised in the long term due to brushing, abrasion, or chemical aging. Therefore, a low initial Ra value alone does not prove the long-term superiority of the coating.

Çakmak et al. compared OptiGlaze and Vita Akzent LC surface coatings with conventional polishing on Crowntec and VarseoSmile Crown Plus materials and showed that surface roughness is affected by both the material and the surface treatment used. In that study, it was reported that conventional polishing produced Ra values similar to or lower than surface coatings in many cases [13]. In our study, however, the fact that OptiGlaze gave the lowest post-polishing numerical values on both resins indicates that the response to surface treatment may depend on the material used, the starting surface, the application method, and the experimental protocol. The differing results between the studies support the idea that a single surface treatment protocol cannot be directly generalized to all permanent 3D-printed resins.

The lowest post-procedural Ra values were descriptively observed in the Optiglaze group. This finding may be explained by the ability of a flowable coating material to fill or mask surface irregularities associated with the layer-by-layer fabrication process, unlike systems that reduce surface defects primarily through mechanical abrasion. In contrast, although TwistDia is a two-step system, it exhibited higher Ra values than the coating-based approach, suggesting that the number of application steps alone may not guarantee a smoother surface. Instead, factors such as abrasive composition, particle size, applied pressure, and polishing duration may play a more decisive role. Similarly, Optishine, a silicon carbide-based single-step brush system, resulted in higher post-procedural Ra values than Optiglaze despite its simpler application protocol. These findings suggest that, in 3D-printed resins, surface-coating approaches may offer an additional advantage in reducing layer-related irregularities and micro-roughness; however, this interpretation should be considered with caution because the overall lowest Ra values were observed in the Lucida group.

Previous studies have shown that polishing paste systems may be effective in reducing surface roughness when used in combination with different paste compositions and application materials [27,28]. One possible explanation for this effect is that polishing pastes contain fine abrasive particles that can reduce surface irregularities during application, thereby creating a more homogeneous surface topography. In addition, paste-based polishing procedures may provide better adaptation to surface irregularities than disc-, brush-, or silicone-based polishing systems [29]. In the study by Bayraktar and colleagues, which evaluated different systems including Lucida DiaShine, surface smoothness improved with polishing in all materials, but the performance of the systems varied depending on the material. This indicates that the compatibility between the hardness and size of the abrasive particles and the matrix and filler properties of the resin may have an effect on the result [15]. In the present study, Lucida, a paste-based polishing system, showed the lowest overall Ra values among the tested finishing and polishing systems. Its Ra values were lower than those of the diamond-impregnated spiral system, TwistDia, and numerically lower than those of the silicon carbide-based brush system, Optishine. This finding suggests that paste-based systems may contribute to the formation of smoother surfaces in 3D-printed resins by providing controlled abrasion and reducing the formation of surface scratches compared with some brush- or spiral-based abrasive systems.

The literature reports varying outcomes regarding surface roughness depending on the abrasive content of polishing systems. In one study using a silicon carbide-coated brush, the surface roughness obtained with the silicon carbide brush was reported to be higher than that achieved with aluminum oxide-containing systems [30]. In another study, however, a polishing system containing diamond particles was found to produce higher surface roughness values than systems containing silicon carbide and aluminum oxide [31]. In the present study, the silicon carbide-based brush system, Optishine, showed the highest post-procedural Ra values descriptively. The diamond-impregnated spiral system, TwistDia, also exhibited higher Ra values than the coating-based system and the polishing paste-containing system. These findings suggest that polishing performance is determined not only by the type of abrasive used, but also by the combined influence of factors such as abrasive particle size and hardness, application steps, restorative material type, operator-related variables, and the initial surface topography of the material.

The differences between our results and previous studies can be attributed to various sources of heterogeneity related to materials and methodology. First, permanent 3D-printed crown resins differ in terms of resin matrix composition, filler content, and surface/mechanical properties, which can affect their response to different polishing procedures [14]. Second, manufacturing parameters and different post-polymerization protocols can contribute to differences in initial surface conditions [14,32,33]. Finally, differences in surface roughness measurement protocols may also have contributed to the variability between reported values [13,14,15]. Therefore, the differing findings in the literature are likely related to the combined effects of material composition, manufacturing and finishing parameters, surface treatment protocol, aging conditions, and measurement methodology, rather than the universal superiority of a single surface treatment system.

The present study has several limitations. In this in vitro study, the specimens had flat surfaces; however, under clinical conditions, restorations present more complex surfaces for finishing and polishing because of their anatomical contours and occlusal morphology. In addition, although all polishing procedures were performed by a single operator using consistent movements parallel to the flat surface at fixed speeds, possible variations in the level of applied pressure during the procedure should be taken into consideration. Therefore, caution is required when directly generalizing the present findings to the clinical setting. Considering these limitations, future studies may evaluate the long-term effects of polishing by incorporating aging protocols that better simulate intraoral conditions. Direct comparison of the results of the present study with those of previous studies is limited, particularly because of substantial differences in study design, especially with respect to the material combinations and polishing protocols used. Further studies involving a greater number of 3D-printing resins and different polishing systems are needed to support the present findings and enhance their generalizability.

## 5. Conclusions

In this study, surface roughness was significantly influenced by the polishing system used. All surface treatment systems reduced surface roughness, while resin type did not significantly affect the overall Ra values. Although all surface treatments provided clinically acceptable surfaces, the combination of resin and finishing–polishing system had an impact on surface roughness. These findings suggest that careful selection of the polishing procedure, together with the restorative material, is essential for optimizing the surface topography of 3D-printed resin restorations.

## Figures and Tables

**Table 1:** Properties of 3D resins used in the study.

Materials	Composition	Manufacturer
VarseoSmile Crown Plus (VSC)	Esterification products of 4,4′-isopropylidiphenol, ethoxylated and 2-methylprop-2enoic acid, silanized dental glass, methylbenzoylformate, diphenyl (2,4,6-trimethylbenzoyl) phosphine oxide, 30–50 wt% inorganic fillers (particle size 0.7 μm)	Bego, Bremen, Germany
Saremco Print Crowntec (SPC)	Esterification products of 4,4′-isopropylidiphenol, ethoxylated and 2-methylprop-2enoic acid, silanized dental glass, pyrogenic silica, initiators. Total content of inorganic fillers (particle size 0.7 μm) is 30–50 wt%.	Saremco Dental AG, Switzerland

**Table 2:** Characteristics of Finishing and Polishing Systems Used in the Study.

Material	Manufacturer	Type	Description
TwistDia	Kuraray, Germany	2 step	Silicon spiral containing 10 and 14 µm diamonds
Lucida	DiaShine, Italy	1 step	Paste containing 1 µm submicron hybrid abrasive
Optishine	Kerr, California, USA	1 step	Silicon carbide polishing particles embedded in the bristles
Optiglaze	GC, Tokyo, Japan	Liquid	Nanofilled light-cured protective coating

**Table 3:** Analysis of factors and interactions using ART ANOVA.

	Test Statistics	*p* ^x^	η*p*^2^
Resin	1.080	0.302	0.015
Polishing system	8.584	<0.001	0.263
Time	464.790	<0.001	0.866
Resin × Polishing system	2.360	0.079	0.090
Resin × Time	0.201	0.655	0.003
Polishing system × Time	2.085	0.110	0.080
Resin × Polishing system × Time	5.226	0.002	0.179

^x^ Art ANOVA; Median was used as the comparison method.

**Table 4:** Surface roughness (Ra, µm) values of polishing systems and time-dependent 3D resins.

Time	Polishing System	Resin		Total
		SPC	VSC	
Pre-polishing	Optiglaze	0.184 (0.16–0.22)	0.183 (0.16–0.22)	0.184 (0.16–0.22)
	Lucida	0.236 (0.15–0.3)	0.179 (0.14–0.21)	0.201 (0.14–0.3)
	Optishine	0.172 (0.14–0.27)	0.215 (0.18–0.48)	0.205 (0.14–0.48)
	TwistDia	0.232 (0.14–0.35)	0.283 (0.14–0.33)	0.265 (0.14–0.35)
	Total	0.198 (0.14–0.35)	0.2 (0.14–0.48)	0.198 (0.14–0.48)
Post-polishing	Optiglaze	0.065 (0.02–0.12)	0.077 (0.04–0.15)	0.07 (0.02–0.15)
	Lucida	0.088 (0.07–0.14)	0.101 (0.05–0.15)	0.095 (0.05–0.15)
	Optishine	0.116 (0.04–0.15)	0.133 (0.07–0.2)	0.126 (0.04–0.2)
	TwistDia	0.126 (0.03–0.18)	0.113 (0.07–0.19)	0.121 (0.03–0.19)
	Total	0.101 (0.02–0.18)	0.102 (0.04–0.2)	0.101 (0.02–0.2)
Total	Optiglaze	0.14 (0.02–0.22)	0.153 (0.04–0.22)	0.153 (0.02–0.22) ^A^
	Lucida	0.147 (0.07–0.3)	0.142 (0.05–0.21)	0.142 (0.05–0.3) ^AB^
	Optishine	0.144 (0.04–0.27)	0.188 (0.07–0.48)	0.15 (0.04–0.48) ^BC^
	TwistDia	0.158 (0.03–0.35)	0.168 (0.07–0.33)	0.159 (0.03–0.35) ^C^
	Total	0.148 (0.02–0.35)	0.156 (0.04–0.48)	

Data are presented as median (minimum–maximum). There is no difference between polishing systems that have the same letter designation.

## Data Availability

The original contributions presented in this study are included in the article. Further inquiries can be directed to the corresponding author.

## Abbreviations

The following abbreviations are used in this manuscript:

3D	Three-dimensional
VSC	VarseoSmile Crown Plus
SPC	Saremco Print Crowntec
